# Sialyllactose in Viral Membrane Gangliosides Is a Novel Molecular Recognition Pattern for Mature Dendritic Cell Capture of HIV-1

**DOI:** 10.1371/journal.pbio.1001315

**Published:** 2012-04-24

**Authors:** Nuria Izquierdo-Useros, Maier Lorizate, F.-Xabier Contreras, Maria T. Rodriguez-Plata, Bärbel Glass, Itziar Erkizia, Julia G. Prado, Josefina Casas, Gemma Fabriàs, Hans-Georg Kräusslich, Javier Martinez-Picado

**Affiliations:** 1 AIDS Research Institute IrsiCaixa, Institut d'Investigació en Ciències de la Salut Germans Trias i Pujol, Universitat Autònoma de Barcelona, Badalona, Spain; 2 Department of Infectious Diseases, Virology, Universitätsklinikum Heidelberg, Heidelberg, Germany; 3 Heidelberg University Biochemistry Center (BZH), Heidelberg; 4 Department of Biomedicinal Chemistry, Institute of Advanced Chemistry of Catalonia (IQAC)/CSIC, Barcelona, Spain; 5 Institució Catalana de Recerca i Estudis Avançats (ICREA), Barcelona, Spain; Fred Hutchinson Cancer Research Center, United States of America

## Abstract

HIV-1 is internalized into mature dendritic cells (mDCs) via an as yet undefined mechanism with subsequent transfer of stored, infectious virus to CD4^+^ T lymphocytes. Thus, HIV-1 subverts a DC antigen capture mechanism to promote viral spread. Here, we show that gangliosides in the HIV-1 membrane are the key molecules for mDC uptake. HIV-1 virus-like particles and liposomes mimicking the HIV-1 lipid composition were shown to use a common internalization pathway and the same trafficking route within mDCs. Hence, these results demonstrate that gangliosides can act as viral attachment factors, in addition to their well known function as cellular receptors for certain viruses. Furthermore, the sialyllactose molecule present in specific gangliosides was identified as the determinant moiety for mDC HIV-1 uptake. Thus, sialyllactose represents a novel molecular recognition pattern for mDC capture, and may be crucial both for antigen presentation leading to immunity against pathogens and for succumbing to subversion by HIV-1.

## Abstract 2

An accessible sialyllactose moiety on viral membrane gangliosides is shown to be essential for HIV-1 uptake into mature dendritic cells, thereby promoting viral transfer and infection of bystander CD4+ T lymphocytes.

## Author Summary

Antigen-presenting cells such as dendritic cells (DCs) are required to combat infections, but viruses including HIV have evolved strategies to evade their anti-viral activity. HIV can enter DCs via a non-infectious endocytic mechanism and trick them into passing infectious virus on to bystander CD4^+^ T cells. Immature DC (iDCs) are characterized by high endocytic activity and low T-cell activation potential. Interestingly, several groups have shown that DCs that have undergone “‘maturation’” (mDCs), a process that occurs on contact with a presentable antigen, capture higher numbers of HIV-1 particles than iDCs when they are matured in the presence of lipopolysaccharide. mDCs move to the lymph nodes where they have more opportunity to interact with T cells than iDCs, and thus to pass on infectious virus. But the molecular mechanism underlying HIV-1 uptake by mDCs has until now been elusive. Here we show that gangliosides, basic components of the host cell's plasma membrane, have an important role in this process. Gangliosides are known to be incorporated into the viral envelope membrane during the process of viral particle budding and here we show that they serve as viral attachment factors: they are recognized and enable HIV-1 uptake by mDCs. Thus, in addition to the well-known function of gangliosides as host cell receptors that mediate virus (e.g., polyoma and SV40) attachment and transport from the plasma membrane to the ER, we now demonstrate that they can also act as determinants for capture by mDCs. Furthermore, we identify a moiety composed of sialyllactose on HIV-1 membrane gangliosides as the specific domain recognized by mDCs. We propose that this novel recognition moiety might be crucial for inducing immune responses, but also critical to disseminate HIV-1 and other ganglioside-containing viruses.

## Introduction

Dendritic cells (DCs) are the most potent antigen-presenting cells found in the organism and play a paramount role in initiating immune responses to assaulting pathogens [1],[2]. DCs that patrol the mucosal tissue display an immature phenotype and are able to capture incoming pathogens, which leads to DC activation, maturation, and migration to the secondary lymphoid tissue, where DCs acquire the mature phenotype required to efficiently induce adaptive immune responses [1].

Given the unique role of DCs in initiating primary immune responses [1], it is generally believed that these antigen-presenting cells are critical to induce resistance to infection [2]–[4]. In the specific case of viral infections, murine DC depletion models have provided in vivo evidence of the DC requirement to induce both humoral and cellular antiviral immune responses [5],[6]. However, some viruses, including HIV-1, have evolved strategies to subvert DC antiviral activity [7],[8]. DCs can even promote HIV-l dissemination, both through the direct release of new virus particles after productive infection, and through transmission of captured viruses to susceptible T cells without DC infection, a process known as *trans*-infection (reviewed in [9]). Direct infection and *trans*-infection occur to a different extent in immature DCs (iDCs) and mature DCs (mDCs) (reviewed in [9],[10]). The initial Trojan horse hypothesis suggested that HIV-1 capture by iDCs in the mucosa may protect the virus from degradation and allow its transport to secondary lymphoid organs, facilitating *trans*-infection of CD4^+^ T cells and fueling viral spread [11],[12]. However, iDCs show rapid degradation of captured viral particles [13]–, and several lines of evidence suggest now that the long-term ability of iDCs to transfer HIV-1 relies on de novo production of viral particles after productive infection [14],[16],[17]. HIV-1 replication in DCs is generally less productive than in CD4^+^ T cells (reviewed in [9],[18]), however, probably due to the presence of cellular restriction factors such as SAMHD1 that limit reverse transcription following viral entry [19],[20].

Maturation of DCs further reduces their ability to support HIV-1 replication [21]–[24], but potently enhances their capacity to *trans*-infect bystander CD4^+^ T cells [15],[21],[25]–[27]. *Trans*-infection occurs via the infectious synapse, a cell-to-cell contact zone that facilitates transmission of HIV-1 by locally concentrating virus and viral receptors [26],[28]. Strikingly, poorly macropinocytic mDCs [29] sequester significantly more complete, structurally intact virions into large vesicles than actively endocytic iDCs [30], and retain greater amounts of virus 48 h post-pulse than iDCs immediately after viral wash [15]. Thus, enhanced mDC *trans*-infection correlates with increased HIV-1 capture and a longer life span of trapped viruses [15],[27]. Furthermore, mDCs efficiently interact with CD4^+^ T cells in lymphoid tissues; key sites of viral replication, where naïve CD4^+^ T cells are activated and turn highly susceptible to HIV-1 infection [31]. Accordingly, carriage of HIV-1 by mDCs could facilitate the loss of antigen-specific CD4^+^ T cells [32],[33], favoring HIV-1 pathogenesis. However, the molecular mechanism underlying HIV-1 uptake by mDCs remains largely uncharacterized.

We have previously identified an HIV-1 gp120-independent mechanism of viral binding and uptake that is upregulated upon DC maturation [15]. Furthermore, HIV-1 Gag enhanced green fluorescent protein (eGFP)–expressing fluorescent virus-like particles (VLP_HIV-Gag-eGFP_) follow the same trafficking route as wild-type HIV-1 in mDCs [34], and hence share a common molecular pattern that governs entry into mDCs. In addition, we also reported that treatment of HIV-1 or VLP producer cells with inhibitors of glycosphingolipid biosynthesis yielded particles with less glycosphingolipids, which exhibited reduced entry into mDCs [34],[35], without affecting net release from producer cells [36]. Thus, we hypothesize that gangliosides in the outer monolayer of HIV-1 and VLP membranes could act as viral attachment factors and allow viral recognition and capture by mDCs.

Here we sought to investigate the molecular determinants involved in viral binding and internalization mediated by mDCs. Using liposomes to mimic the lipid composition and size of HIV-1, we demonstrate that gangliosides are the key molecules that mediate liposome uptake. We extended these observations to VLPs and HIV-1, characterizing a new role for these glycosphingolipids as viral attachment factors. Furthermore, we identify sialyllactose on HIV-1 membrane gangliosides as a novel molecular recognition pattern that mediates virus uptake into mDCs.

## Results

### Gangliosides Are Required for Viral Capture Mediated by mDC

**Figure 1:**
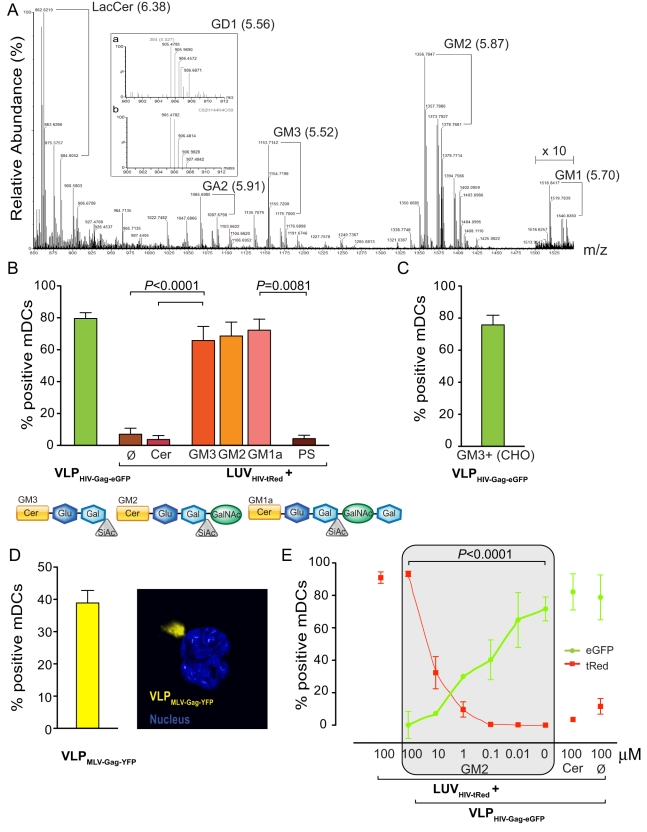
Gangliosides are required for viral capture mediated by mDC. (A) Ganglioside detection in lipid extracts from MT4 derived HIV_NL4-3_.Partial mass spectrum (from 850 to 1550 amu) corresponding to the 5.3- and 6.5-min range of a UPLC/TOF ESI (+) chromatogram representative of three different viral isolations. The selected time range corresponds to the *N*-hexadecanoyl (N-C16) species. The N-C22, N-C24, and N-C24:1 species were also observed. For each compound of interest identified, the exact mass of its [M+H]+ and [M+Na]+ ions are indicated. The retention time of each compound is given within parenthesis next to its abbreviation. Inset: exact mass ion cluster obtained at 5.56 min for GD1 (a) and exact mass ion cluster corresponding to the formula C82H144N4O39 with a charge state of 2 (b). X 10 indicates a ten-fold magnification between 1,500 and 1,550 amu. GA2, *N*-acetyl-D-galactosaminyl-D-galactosyl-D-glucosylceramide; LacCer, D-galactosyl-D-glucosylceramide (lactosylceramide). (B) Comparative mDC capture of VLP_HIV-Gag-eGFP_ produced in HEK-293T and distinct fluorescent LUV_HIV-tRed_ containing Cer, GM3, GM2, GM1a, or PS. A total of 2×10^5^ DCs were pulsed for 4 h at 37°C with 100 µM of LUV or 75 ng of VLP_HIV-Gag-eGFP_ Gag in 0.2 ml, washed with PBS, and assessed by FACS to obtain the percentage of tRed or eGFP-positive cells. Data show mean values and standard error of the mean (SEM) from five independent experiments including cells from at least six donors. mDCs capture significantly higher amounts of GM3-containing LUV_HIV-tRed_ than Cer or Ø LUV_HIV-tRed_ (*p*<0.0001, paired *t* test). mDCs capture significantly higher amounts of GM1a-containing LUV_HIV-tRed_ than negatively charged PS-LUV_HIV-tRed_ (*p* = 0.0081, paired *t* test). Schematic representation of the gangliosides used in the LUVs for these experiments is shown underneath. (C) Capture of VLP_HIV-Gag-eGFP_ produced in CHO cell line, which is only able to synthesize gangliosides up to GM3. A total of 2×10^5^ mDCs were incubated for 4 h at 37°C with 75 ng of sucrose-pelleted VLP_HIV-Gag-eGFP_ Gag, washed and analyzed by FACS to determine the percentage of eGFP-positive cells. Data show mean values and SEM from one representative experiment out of two including cells from three donors. (D) mDC capture of VLP_MLV-Gag-YFP_. Graph shows mDCs pulsed for 4 h at 37°C with VLP_MLV-Gag-YFP_, washed with PBS and assayed by FACS to obtain the percentage of YFP-positive cells. Data show mean values and SEM of cells from three donors. Left image depicts **c**onfocal microscopy analysis of pulsed cells, showing a 3-D reconstruction of the *x*-*y* sections collected throughout the whole mDC *z* volume every 0.1 µm. Isosurface representation of DAPI-stained nucleus is depicted, computing the maximum intensity fluorescence of the sac-like compartment surface within a 3-D volumetric *x*-*y*-*z* data field, where VLP_MLV-Gag-YFP_ accumulate within a compartment analogous to that observed for HIV-1. (E) Capture competition between 75 ng of VLP_HIV-Gag-eGFP_ Gag and decreasing amounts (µM) of GM2-containing LUV_HIV-tRed_. As controls, we used the maximum concentration of LUV_HIV-tRed_ (100 µM) with or without Cer. Cells were incubated for 4 h at 37°C, washed and analyzed by FACS to establish the percentage of eGFP- and tRed-positive cells. Data show mean values and SEM from three independent experiments including cells from at least four donors. mDCs capture fewer VLP_HIV-Gag-eGFP_ in the presence of higher amounts of GM2-containing LUV_HIV-tRed_ (*p*<0.0001, paired *t* test).

Considering that glycosphingolipids are enriched in raft-like plasma membrane domains [37]–[39] from where HIV-1 is thought to bud (reviewed in [40]), we investigated the potential role of glycosphingolipids for HIV-1 capture by mDCs. The ganglioside GM3 was previously identified in the membrane of different retroviruses including HIV-1 [41],[42]. We were able to confirm the presence of GM3 in HIV_NL4-3_ derived from the T-cell line MT-4 by mass spectrometry (Figure 1A). In addition, we detected several other gangliosides including GM1, GM2, and GD1 in the HIV-1 membrane (Figure 1A).

To test whether gangliosides in the outer leaflet of HIV-1 or vesicular membranes can act as attachment factors yielding mDC uptake, we prepared Texas Red (tRed)-labeled large unilamellar vesicles (LUV) mimicking the size and lipid composition of HIV-1 (referred to as LUV_HIV-tRed_ and prepared as in [43]) and containing different gangliosides (Figure S1). All LUVs displayed equal fluorescence intensities (Figure S2). mDCs were pulsed with either LUV_HIV-tRed_ or VLPs for 4 h at 37°C and the percentage of fluorescent cells was determined by fluorescence activated cell sorting (FACS). Similar to our previous results with infectious HIV-1 [15], a high percentage of mDCs captured the fluorescent VLP_HIV-Gag-eGFP_ (Figure 1B). Furthermore, VLPs produced in the CHO cell line, which is only able to synthesize gangliosides up to GM3 [44], were also efficiently captured by mDCs (Figure 1C). Uptake into mDCs was further observed for the murine retrovirus MuLV (Figure 1D), which was previously shown to also contain gangliosides [41]. On the other hand, no significant uptake into mDCs was observed for LUV_HIV-tRed_, which contained the main lipid constituents of HIV-1, but were devoid of gangliosides (*p*<0.0001, paired *t* test) (Figures 1B and S3). Uptake into mDCs remained negative for LUV_HIV-tRed_ containing ceramide (Cer) (*p*<0.0001, paired *t* test) (Figures 1B and S3). This was completely different when monosialogangliosides such as GM3, GM2, or GM1a were incorporated into the LUVs; mDCs were able to capture these liposomes with equal efficiency as VLP_HIV-Gag-eGFP_ (Figures 1B and S3). To ensure that this capture was not merely due to electrostatic interactions between negatively charged gangliosides and surface charges on mDCs, LUV_HIV-tRed_ containing negatively charged phosphatidylserine (PS) were analyzed in parallel and were found to be negative for mDC capture (*p* = 0.0081, paired *t* test) (Figure 1B). These results reveal that monosialogangliosides mediate LUV capture by mDCs, and that the carbohydrate head group is essential for this process.

To determine whether ganglioside-containing LUV_HIV-tRed_ and VLP_HIV-Gag-eGFP_ exploit a common entry mechanism into mDCs, we performed competition experiments. mDCs were pulsed with decreasing amounts of GM2-containing LUV_HIV-tRed_ and a constant amount of VLP_HIV-Gag-eGFP_ for 4 h at 37°C. After extensive washing, the percentage of eGFP- and tRed-positive cells was determined by FACS. GM2-containing LUV_HIV-tRed_ efficiently competed for the uptake of VLP_HIV-Gag-eGFP_ into mDCs in a dose-dependent manner (*p*<0.0001, paired *t* test) (Figure 1E). However, no competition for VLP uptake was observed for LUV_HIV-tRed_ containing Cer or lacking glycosphingolipids (Figure 1E). Hence, GM-containing LUV_HIV-tRed_ and VLP_HIV-Gag-eGFP_use a common entry mechanism to gain access into mDCs, which is dependent on the carbohydrate head group.

### Ganglioside-Containing LUV_HIV-tRed_ Traffic to the Same Compartment as VLP _HIV-Gag-eGFP_ in mDCs

**Figure 2:**
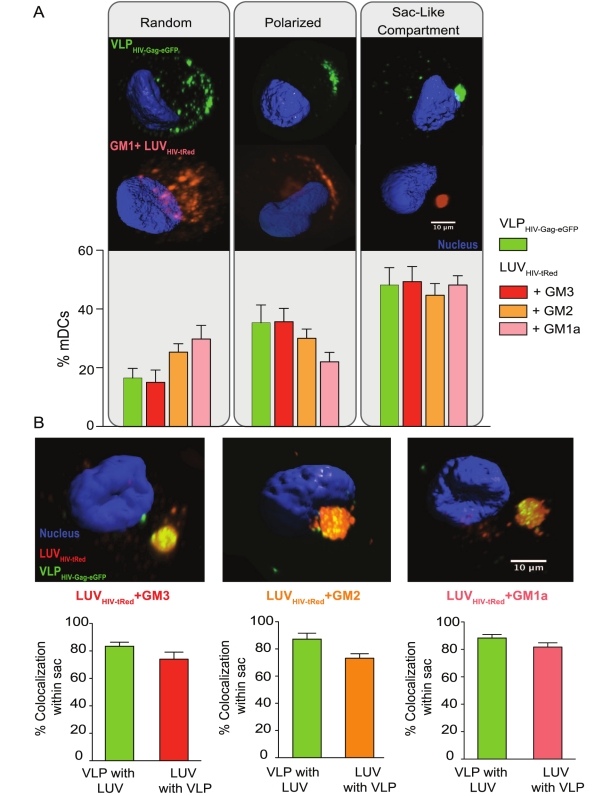
Ganglioside-containing LUV_HIV-tRed_ traffic to the same compartment as VLP _HIV-Gag-eGFP_ in mDCs. (A) (Top) Fluorescence images showing the different patterns of 75 ng of VLP_HIV-Gag-eGFP_ Gag and 100 µM of GM1a-containing LUV_HIV-tRed_ capture mediated by mDCs: random binding, polarized accumulation, and formation of a sac-like compartment. 3-D reconstructions of the *x*-*y* sections were performed collecting images every 0.1 µm throughout the whole mDC volume. Isosurface representation of DAPI-stained nucleus is shown, computing the maximum intensity of the green and red fluorescence within a 3-D volumetric *x*-*y*-*z* data field. (Bottom) Percentage of mDCs with distinct capture patterns after 4 h of independent challenging with VLP_HIV-Gag-eGFP_ or ganglioside-containing LUV_HIV-tRed_. Cells were classified using confocal microscopy displaying particles as indicated in the top images. Data show mean values and SEM of more than 100 cells from five different donors. (B) Confocal microscopy analysis of mDCs previously pulsed with 100 µM of GM1a, GM2, and GM3 containing LUV_HIV-tRed_ and then exposed to 75 ng of VLP_HIV-Gag-eGFP_ Gag. (Top images) 3-D reconstructions of the *x*-*y* sections collected throughout the whole mDC *z* volume every 0.1 µm. Isosurface representation of DAPI stained nucleus is shown, computing the maximum intensity fluorescence of the sac-like compartment surface within a 3-D volumetric *x*-*y*-*z* data field, where VLP_HIV-Gag-eGFP_ and ganglioside-containing LUV_HIV-tRed_ are accumulated within the same compartment. (Bottom graphs) Quantification of the percentage of VLP_HIV-Gag-eGFP_ co-localizing with ganglioside-containing LUV_HIV-tRed_ and *vice versa*, obtained analyzing at least ten compartments from mDCs of three different donors. The mean and standard deviation of the thresholded correlation coefficients of Manders and Pearson (obtained considering all the images) were 0.752±0.03 and 0.44±0.11, respectively, indicating co-localization.

We next investigated whether GM-containing LUV_HIV-tRed_ and VLP_HIV-Gag-eGFP_ reach the same compartment in mDCs using spinning-disc confocal microscopy. We had previously described three types of patterns for HIV-1 captured into mDC: random, polarized, or sac-like compartments [15],[45]. The same patterns were also observed for GM-containing LUV_HIV-tRed_ and the percentage of mDCs displaying the different patterns was similar regardless of the particle used (Figure 2A). Thus, VLP_HIV-Gag-eGFP_ and GM-containing LUV_HIV-tRed_ not only compete for internalization, but also traffic to an analogous compartment within mDCs. To determine whether VLP_HIV-Gag-eGFP_ and GM-containing LUV_HIV-tRed_ are captured into the same compartment, mDCs were pre-incubated 3 h at 37°C with GM-containing LUV_HIV-tRed_ and subsequently incubated with VLP_HIV-Gag-eGFP_ for three additional hours. Confocal microscopy of fixed cells revealed that GM-containing LUV_HIV-tRed_ and VLPs polarized towards the same cell area in mDCs (Figure S4). Furthermore, VLPs extensively co-localized with GM-containing LUV_HIV-tRed_ (including either GM1a, GM2, or GM3) in the same intracellular compartment (Figure 2B; Video S1).

### Capture of Ganglioside-Containing LUV_HIV-tRed_ by mDCs Is Independent of Membrane Liquid Order

**Figure 3:**
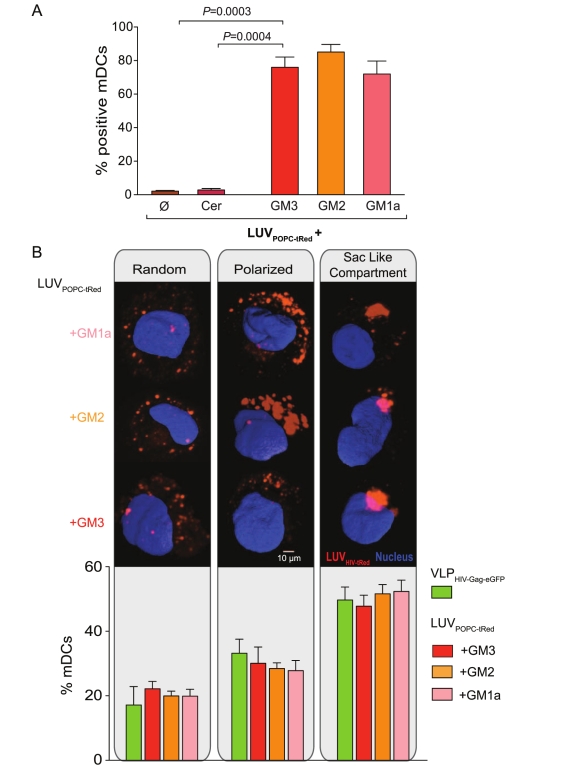
Capture of ganglioside-containing LUV_HIV-tRed_ by mDCs is independent of membrane liquid order. (A) Comparative mDC capture of LUV_POPC-tRed_ containing or not Cer, GM3, GM2, or GM1a. A total of 2×10^5^ DCs were pulsed for 4 h at 37°C with 100 µM of LUVs, washed with PBS, and assessed by FACS to obtain the percentage of tRed-positive cells. Data show mean values and SEM from three independent experiments including cells from at least six donors. mDCs capture significantly higher amounts of GM3-containing LUV_POPC-tRed_ than Cer or LUV_POPC-tRed_ (*p*-values on the graph, paired *t* test). (B) (Top images) Fluorescence images showing the different phases of ganglioside-containing LUV_POPC-tRed_ capture mediated by mDCs: random binding, polarized accumulation, and formation of a sac-like compartment. 3-D reconstructions of the *x*-*y* sections were performed collecting images every 0.1 µm throughout the whole mDC *z* volume. Isosurface representation of DAPI stained nucleus is shown, computing the maximum intensity of the green and red fluorescence within a 3-D volumetric *x*-*y*-*z* data field. (Bottom graph) Percentages of mDCs with distinct liposome capture pattern after 4 h of ganglioside-containing LUV_POPC-tRed_ challenging. Cells were classified using confocal microscopy displaying particles as indicated in the top images. Data show mean values and SEM of more than 100 mDCs from three different donors.

The HIV-1 envelope is a liquid-ordered membrane and gangliosides are presumably enriched in this type of membranes [41],[43],[46],[47]. Moreover, ganglioside interaction with cholesterol in lipid rafts [37],[38],[47] is known to influence ganglioside conformation and alter its activity as a cellular receptor [48]. We therefore assessed whether membrane structure or the specific lipid composition (other than gangliosides) of the particle membrane influenced mDC capture. mDCs were incubated with LUV_POPC-tRed_composed of 1-palmitoyl-2-oleoyl-sn-glycero-3-phosphocholine (POPC) with or without different gangliosides (Figure 3A). In contrast to LUV_HIV-tRed_, LUV_POPC-tRed_ have a liquid-disordered membrane structure [38]. Results for LUV_POPC-tRed_ were very similar to LUV_HIV-tRed_ with efficient capture if either GM1a, GM2, or GM3 was present, while no uptake was observed for Cer containing LUV_POPC-tRed_ or LUV_POPC-tRed_ lacking gangliosides (Figure 3A). Furthermore, the percentage of mDCs displaying particles in random, polarized or sac-like compartment capture patterns was again very similar for the different particles (Figure 3B). These results show that ganglioside-containing LUVs use the same trafficking pathway as VLP_HIV-Gag-eGFP_ regardless of their membrane structure, indicating that gangliosides themselves are the key molecules responsible for mDC capture.

### Ganglioside Complexity Determines mDC Capture

**Figure 4:**
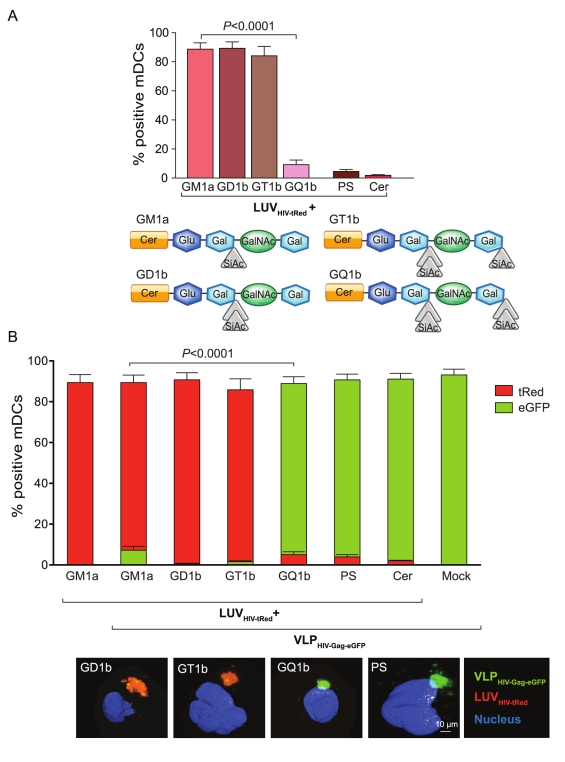
Ganglioside complexity determines mDC capture. (A) Comparative mDC capture of distinct LUV_HIV-tRed_ containing GM1a, polysialogangliosides such as GD1b, GT1b, and GQ1b; PS and Cer. A total of 2×10^5^ DCs were pulsed for 4 h at 37°C with 100 µM of LUVs, washed with PBS, and assessed by FACS to obtain the percentage of tRed-positive cells. Data show mean values and SEM from two independent experiments including cells from six donors. mDCs capture significantly higher amounts of GM1a-containing LUV_HIV-tRed_ than GQ1b-containing LUV_HIV-tRed_ (*p*<0.0001, paired *t* test). Schematic of the gangliosides in the LUVs employed for these experiments is shown underneath. (B) Capture competition between mDCs pulsed with 75 ng of VLP_HIV-Gag-eGFP_ Gag and 100 µM of different polysialogangliosides LUV_HIV-tRed_. Cells were incubated for 4 h at 37°C, washed, and analyzed by FACS to determine the percentage of eGFP- and tRed-positive cells. Data show mean values and SEM from two independent experiments including cells from six donors. mDCs capture fewer VLP_HIV-Gag-eGFP_ in the presence of GM1a-containing LUV_HIV-tRed_ than in the presence of the same concentration of GQ1b containing LUV_HIV-tRed_ (*p*<0.0001, paired *t* test). Representative fluorescence images from mDCs exposed to the indicated LUV_HIV-tRed_ in these experiments are shown in the bottom of the graph. Isosurface representation of DAPI stained nucleus is shown, computing the maximum intensity of the green and red fluorescence within a 3-D volumetric *x*-*y*-*z* data field.

To gain further insight into the molecular determinant structure required for efficient recognition by mDCs, LUV_HIV-tRed_ carrying more complex gangliosides were produced, including two, three, and four sialic acid groups at diverse positions in the carbohydrate polar head group (di-, tri-, and tetra-sialogangliosides) (Figure 4A). mDCs pulsed with an equal amount of LUV_HIV-tRed_-containing gangliosides with two or three sialic acids (GD1b and GT1b, respectively) captured these particles with the same efficiency as GM1a-LUV_HIV-tRed_ (Figure 4A). However, capture was almost completely lost for LUV_HIV-tRed_ containing a ganglioside with four sialic acids (GQ1b) (Figure 4A). Accordingly, LUV_HIV-tRed_ carrying GD1b or GT1b efficiently competed for mDC uptake with VLP_HIV-Gag-eGFP_, while no competition was observed for LUV_HIV-tRed_ carrying GQ1b, PS, or Cer (Figure 4B). These results indicate that complex gangliosides with up to three sialic acids located in distinct positions of the carbohydrate head group share a common structure determinant for mDC uptake, which is lost in GQ1b.

### Identification of the Molecular Recognition Domain Present in Gangliosides That Is Essential for mDC Capture

**Figure 5:**
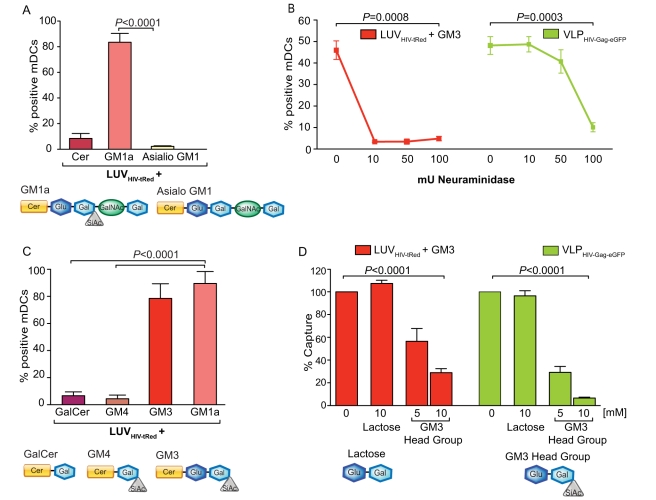
Identification of the molecular recognition domain present in gangliosides essential for mDC capture. (A) Comparative mDC capture of distinct LUV_HIV-tRed_ containing Cer, GM1a, or GM1 lacking sialic acid (Asialo GM1). A total of 2×10^5^ DCs were pulsed for 4 h at 37°C with 100 µM of LUVs, washed with PBS, and assessed by FACS to obtain the percentage of tRed-positive cells. Data show mean values and SEM from three independent experiments, including cells from nine donors. mDCs capture significantly higher amounts of GM1a-containing LUV_HIV-tRed_ than Asialo GM1-containing LUV_HIV-tRed_ (*p*<0.0001, paired *t* test). Schematic of the gangliosides present in the LUVs of these experiments is shown underneath. (B) Comparative mDC capture of GM3-containing LUV_HIV-tRed_ and VLP_HIV-Gag-eGFP_ treated or not with neuraminidase to remove sialic acid. A total of 2×10^5^ DCs were pulsed for 2 h at 37°C with 25 µM of LUVs and 75 ng of VLP_HIV-Gag-eGFP_ Gag treated or not with *C. perfringens* neuraminidase for 12 h, washed with PBS, and assessed by FACS to obtain the percentage of tRed- and eGFP-positive cells. Data show mean values and SEM from two independent experiments including cells from five donors. mDCs capture significantly higher amounts of untreated particles than neuraminidase-treated particles (*p-*values on the graph, paired *t* test). (C) Comparative mDC capture of distinct LUV_HIV-tRed_ containing GalCer, GM4, GM3, or GM1a. A total of 2×10^5^ DCs were pulsed for 4 h at 37°C with 100 µM of LUVs, washed, and assessed by FACS to obtain the percentage of tRed-positive cells. Data show mean values and SEM from three independent experiments including cells from nine donors. mDCs capture significantly higher amounts of GM1a-containing LUV_HIV-tRed_ than GalCer or GM4-containing LUV_HIV-tRed_ (*p*<0.0001, paired *t* test). Schematic of the molecules present in the LUVs for these experiments is shown underneath. (D) Graph representing the relative capture of GM3-containing LUV_HIV-tRed_ and VLP_HIV-Gag-eGFP_ by mDCs that had been pre-incubated with 10 mM of soluble lactose or with 5–10 mM of GM3 carbohydrate polar head group, normalized to the level of LUV/VLP capture by mock-treated mDCs (set at 100%). mDCs captured fewer particles upon treatment with GM3 polar head group (*p-*values on the graph, paired *t* test). Data show mean values and SEM from three independent experiments including cells from at least nine donors.

The lack of internalization of Cer-containing LUVs indicated that the carbohydrate head group is specifically required for mDC capture. Sialic acid has previously been identified as a cellular receptor for certain viruses [49]. We therefore tested its importance for mDC capture. Incubation of mDCs with equal concentrations of LUV_HIV-tRed_ containing Cer, GM1a, or GM1 without the sialic acid group (Asialo GM1) revealed sialic acid-dependent capture (Figure 5A). In addition, in situ neuraminidase treatment of GM3-containing LUV_HIV-tRed_ and VLP_HIV-Gag-eGFP_, significantly reduced particle capture (Figure 5B) and LUV binding to mDCs (Figure S5). Thus, the sialic acid moiety in gangliosides is necessary for specific recognition by mDCs. To assess the contribution of other components of the carbohydrate head group, we prepared LUV_HIV-tRed_ containing either GM4 (lacking the glucose moiety of GM3) (Figure 5C) or GalCer (lacking both the glucose and sialic acid moieties of GM3) (Figure 5C). mDCs incubated with GM4- or GalCer-containing LUV_HIV-tRed_ showed only background levels of liposome capture (Figure 5C), indicating that the glucose moiety of sphingolipids is also necessary for mDC capture.

Given that the carbohydrate moiety within gangliosides constitutes the molecular recognition determinant for mDC capture, these head groups should compete for VLP and LUV uptake. Capture of GM3-containing LUV_HIV-tRed_ or VLP_HIV-Gag-eGFP_ by mDCs was completely blocked in the presence of the GM3 polar head group (sialyllactose), while equal concentrations of lactose (lacking the sialic acid group) had no effect (Figure 5D). Taken together, these data clearly show that the sialyllactose moiety of gangliosides is the molecular determinant required for efficient VLP and LUV recognition and capture by mDCs. Noteworthy, the high concentrations of the GM3 head group required for competition in Figure 5D compared to the low concentrations of gangliosides in LUVs needed to outcompete VLPs (∼1,000-fold less) (Figure 1C) suggests that the attachment of sialyllactose to Cer within membranes confers a higher binding avidity. This is not surprising since viruses and toxins are multivalent: for instance, the VP1 capsid protein of SV40 is pentameric and the cholera toxin is pentavalent; both bind five GM1 molecules [50]–[52]. In addition, the hydrophilic moiety of Cer in the membrane interface could be part of the recognition domain, directly increasing the binding affinity to mDCs.

Modeling of the 3-D structure of gangliosides (Figure S6) suggested that mDC recognition required an exposed sialyllactose domain and that the lack of recognition of GQ1b could be caused by steric hindrance (Figure S6). Thus, although the lipid and protein context of surrounding membranes might influence the biologically active conformation of gangliosides [48],[53]; 3-D reconstructions provide a structural basis to explain the distinct recognition patterns observed for different gangliosides. The sialyllactose recognition domain defined in this study differs from the NeuAc α 2,3Gal β 1,3GalNAc on gangliosides identified as host cell receptors for SV40 and Sendai virus, and for cholera and tetanus toxin [50],[52],[54], but is identical with the α 2,3-sialyllactose identified as host cell receptor for other paramyxoviruses [55],[56].

### Sialyllactose in Membrane Gangliosides Is Required for mDC Uptake and *Trans*-infection of HIV-1

**Figure 6:**
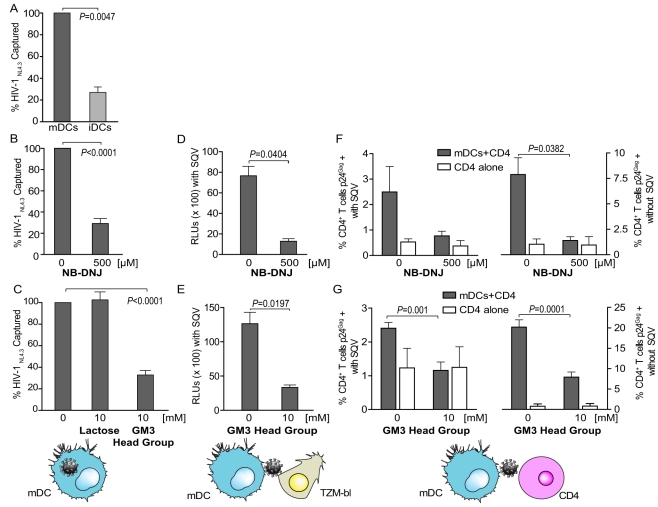
Sialyllactose in membrane gangliosides is required for mDC uptake and *trans*-infection of HIV-1. (A) Relative capture of HIV_NL4-3_ produced in primary cells by mDCs and iDCs. DCs were pulsed for 4 h at 37°C with equal amounts of a HIV_NL4-3_ produced in stimulated PBMCs, extensively washed, and then assayed for cell-associated p24^Gag^ content by ELISA. Results are expressed as the percentage of HIV_NL4-3_ captured by iDCs relative to mDCs, normalized to 100% of viral capture. Viral uptake was increased in mDCs compared to iDCs (*p* = 0.0047, one sample *t* test). Data show mean values and SEM from two independent experiments including cells from three donors. (B) Relative capture of HIV_NL4-3_ produced in CD4^+^ T cells that had been treated or not with the glycosphingolipid inhibitor NB-DNJ. mDCs were pulsed for 4 h at 37°C with equal amounts of HIV_NL4-3_ generated in NB-DNJ-treated or mock-treated CD4^+^ T cells and then assayed for p24^Gag^ content. mDCs captured less HIV_NL4-3_ generated in NB-DNJ-treated CD4^+^ T cells (*p*<0.0001, one sample *t* test). Data show mean values and SEM from two independent experiments including cells from six donors. (C) Competition of HIV_NL4-3_ capture by mDCs in the presence of the GM3 head group. mDCs were either pre-incubated with 10 mM lactose, 10 mM GM3 head group, or mock treated, and were then pulsed for 4 h at 37°C with equal amounts of HIV_NL4-3_ generated in CD4^+^ T cells. Virus uptake was assayed by ELISA for p24^Gag^. HIV_NL4-3_ capture was strongly reduced upon pre-treatment with the GM3 head group, but not lactose (*p*<0.0001, one sample *t* test). Data show mean values and SEM from two independent experiments including cells from six donors. (D) Transmission of HIV_NL4-3_ produced in NB-DNJ or mock-treated CD4^+^ T cells to the TZM-bl target cell line, which expresses the luciferase gene under control of the HIV LTR. mDCs treated as described in (B) but pulsed with an equal MOI of 0.1 were extensively washed and co-cultured with TZM-bl cells in the presence of saquinavir (to prevent second round infection) for 48 h before measurement of luciferase activity. *Trans*-infection was less efficient for HIV_NL4-3_ generated in NB-DNJ-treated compared to untreated CD4^+^ T cells (*p* = 0.0404, paired *t* test). Data show mean values and SEM from one experiment including cells from three donors. (E) Competition of *trans*-infection of HIV_NL4-3_ in the presence of the GM3 head group. mDCs treated as described in (C) but pulsed with an MOI of 0.1 were extensively washed and co-cultured with TZM-bl in the presence of saquinavir for 48 h before measurement of luciferase activity. *Trans*-infection was significantly reduced by the GM3 head group (*p* = 0.0197, paired *t* test). Data show mean values and SEM from two independent experiments including cells from six donors. (F) *Trans*-infection of activated primary CD4^+^ T cells by co-culture with mDCs pulsed with HIV_NL4-3_ produced in NB-DNJ or mock- treated CD4^+^ T cells. mDCs were pulsed with HIV_NL4-3_ at an equivalent MOI of 0.1 as described in (B). Unwashed pulsed mDCs were subsequently co-cultured with primary CD4^+^ T cells for 48 h before measuring the intracellular p24^Gag^ content in the lymphocyte gate (CD2^+^ and CD11c^−^ cells) by FACS (filled bars). Equivalent amounts of cell-free HIV_NL4-3_ were used as a control (open bars). Experiments were done in the presence (left panel) or absence (right panel) of saquinavir to distinguish net *trans*-infection from re-infection events, respectively. mDCs transferred less HIV_NL4-3_ generated in NB-DNJ-treated CD4^+^ T cells (*p* = 0.0382, paired *t* test). Data show mean values and SEM from one representative experiment including cells from three donors. (G) *Trans*-infection of activated primary CD4^+^ T cells by co-culture with mDCs pulsed with HIV_NL4-3_ in the presence or absence of the GM3 head group. mDCs were pulsed with HIV_NL4-3_ at an equivalent MOI of 0.1 as described in (C). Unwashed pulsed mDCs were subsequently co-cultured with primary CD4^+^ T cells for 48 h before measuring the intracellular p24^Gag^ content in the lymphocyte gate (CD2^+^ and CD11c^−^ cells) by FACS, as detailed in (F). mDCs transferred less HIV_NL4-3_ upon treatment with the GM3 head group (*p-*values on the graph, paired *t* test). Data show mean values and SEM from two independent experiments including cells from six donors.

To determine whether the results obtained with LUVs and VLPs also hold true for the authentic virus, we performed experiments with wild-type HIV_NL4-3_ produced in primary T cells. Similar to our previous data [15], a high percentage of mDCs captured HIV-1, while uptake into iDCs was much less efficient (*p* = 0.0047, one sample *t* test) (Figure 6A). To confirm the importance of viral gangliosides for mDC capture, we purified HIV_NL4-3_ from primary CD4^+^ T cells pre-treated or not with the glycosphingolipid biosynthesis inhibitor NB-DNJ. HIV-1 capture was strongly reduced for virus obtained from inhibitor-treated cells compared to control virus (*p*<0.0001, one sample *t* test) (Figure 6B). To directly determine the importance of the sialyllactose head group for mDC capture of authentic HIV-1, we performed competition experiments showing a strong reduction of virus capture in the presence of the GM3 polar head group, but not in the presence of lactose (*p*<0.0001, one sample *t* test) (Figure 6C). These data confirm the observations obtained with liposomes and VLPs for authentic HIV-1 from primary CD4^+^ T cells.

HIV-1 capture by mDCs has been shown to promote *trans*-infection of CD4^+^ T cells and other target cells [15],[21],[25]–[27], and we therefore analyzed whether sialyllactose recognition by mDCs is also important for viral transmission. Co-culturing mDCs that had been exposed to an equivalent amount of infectious HIV_NL4-3_ derived from NB-DNJ-treated or untreated CD4^+^ T cells with the TZM-bl reporter cell line revealed a strong reduction of *trans*-infection for the virus from inhibitor-treated cells compared to control virus (*p* = 0.0404, paired *t* test) (Figure 6D). We also observed a strong reduction of *trans*-infection for mDCs pulsed with HIV_NL4-3_ in the presence of the GM3 polar head group and subsequently incubated with TZM-bl cells (Figure 6E) (*p* = 0.0197, paired *t* test). These results were further confirmed when we co-cultured HIV-1-pulsed mDCs with activated primary CD4^+^ T cells. Co-cultures were performed in the presence or absence of the protease inhibitor saquinavir to distinguish net *trans*-infection from re-infection events (Figure 6F and 6G; left and right panels, respectively). Infection of primary CD4^+^ T cells was strongly enhanced when they were co-cultured with HIV-1 pulsed mDCs (Figure 6F and 6G; filled bars). This effect was abrogated when mDCs were pulsed with virus produced from NB-DNJ-treated cells (Figure 6F) or cultured with the GM3 polar head group (Figure 6G). These data indicate that the sialyllactose moiety of gangliosides is the molecular determinant required for efficient HIV-1 capture by mDCs and for subsequent viral *trans*-infection.

## Discussion

Sialic acid on gangliosides has previously been shown to function as host cell receptor for several viruses [54],[57],[58] and for human toxins [52]. The current study clearly identifies a novel role for sialylated gangliosides in the membrane of viruses or LUVs as determinants for specific capture by mDCs. This recognition is reminiscent of the engulfment of apoptotic cells by phagocytes such as DCs, which is triggered by PS, a phospholipid normally found in the inner leaflet of the plasma membrane of living cells, but exposed on the surface of dying cells [59]. However, the viral capture described here is dependent on an exposed sialyllactose moiety on gangliosides, which we identified as a novel molecular recognition pattern. The ganglioside GM3 was previously detected in the membrane of HIV-1 and several other viruses (SFV, VSV, MuLV) [41],[42], and this was extended to GM1, GM2, and GD1 for HIV-1 in the present study. Gangliosides are significant components of the plasma membrane lipidome [42], suggesting that all enveloped viruses, which bud from the plasma membrane of infected cells, may be captured into mDCs by the reported mechanism unless they exclude sialyllactose-containing gangliosides. Most studies of viral lipidomes have not included gangliosides so far, however, and it will be important to determine whether certain viruses have developed mechanisms to prevent sialyllactose presentation on their membrane lipids. This would be conceivable for influenza virus, which carries a viral neuraminidase needed for removal of the sialic acid receptor from the producer cell surface, thus allowing virus release. This neuraminidase may also remove sialic acid groups from viral gangliosides, thus preventing mDC uptake and potential antigen presentation. Consequently, neuraminidase inhibitor treatment should lead to increased DC capture and potentially enhance immunogenicity of influenza virus or VLPs. Moreover, viral ganglioside content may vary depending on the membrane composition of the producer cell. Viral replication in the nervous system, where gangliosides are particularly enriched [60], may thus lead to the insertion of an increased amount of distinct gangliosides into virions, affecting mDC recognition and local immunosurveillance.

The potential immunological role of mDC uptake implies efficient antigen capture and processing throughout the antigen presentation pathway. Interestingly, antigen-bearing cellular secreted vesicles known as exosomes also follow the same trafficking route as HIV-1 [34] and contain gangliosides such as GM3 or GM1 [61]. Hence, sialyllactose-carrying gangliosides in the membrane of viruses and cellular vesicles are targeting molecules for mDC uptake, a pathway that may normally lead to antigen processing and presentation and has been subverted by HIV-1 for infectious virus storage and transmission. Furthermore, although downregulation of endocytosis is considered a hallmark of DC maturation (reviewed in [3]), there is increasing evidence that under inflammatory conditions mDCs capture, process, and present antigens without exclusively relying on prior pathogen exposure [62],[63]. This scenario might be particularly relevant in chronic infections, such as the one caused by HIV-1, where translocation of bacteria from the intestinal lumen [64] could stimulate DCs systemically and contribute to sustained antiviral immune responses. Remarkably, HIV-1 infected patients show enhanced GM3 content in the plasma membrane of T lymphocytes and high titers of anti-lymphocytic GM3 antibodies [65],[66].

Paradoxically, HIV-1 capture into mDCs appears to also critically enhance viral dissemination in lymphoid tissue by efficient release of infectious virus to CD4^+^ T cells in the DC-T-cell synapse, thus promoting pathogenesis and disease progression through *trans*-infection. Hence, although myeloid cells are largely refractory to productive HIV-1 infection due to the presence of cellular restriction factors such as the recently identified SAMHD1 [19],[20], the sialyllactose driven *trans*-infection process characterized here in mDCs seems to exploit a pre-existing cellular-trafficking machinery that avoids the activation of these intrinsic immune pathways.

The efficient capture of ganglioside-carrying cellular vesicles or virions suggests a model where a specific receptor present on the cell surface of mDCs (and possibly other cells) recognizes the sialyllactose moiety on virions or vesicle membranes. Gangliosides have been reported to function as cell adhesion molecules [67], and this may also involve such a receptor. Specific recognition of vesicular gangliosides would then trigger uptake of the respective particles into an intracellular compartment from where they are either recycled to the surface (as in HIV-1 transmission to CD4^+^ T cells) or fed into the antigen presentation pathway. The results of the current study identify sialyllactose on membrane gangliosides as the relevant molecular recognition pattern, explaining the specificity of this process and providing the basis for its future exploitation for interventional or vaccine purposes.

## Materials and Methods

### Ethics Statement

The institutional review board on biomedical research from Hospital Germans Trias i Pujol approved this study.

### Isolation of HIV-1 and Mass Spectrometry Analysis

MT-4 cells were infected with HIV_NL4-3_ and co-cultured with uninfected cells. Virus was harvested before cytopathic effects were observed, and purified essentially as described in [43]. Briefly, medium was cleared by filtration, and particles were concentrated by ultracentrifugation through a cushion of 20% (w/w) sucrose. Concentrated HIV-1 was further purified by velocity gradient centrifugation on an OptiPrep gradient (Axis-Shield). The visible virus fraction was collected and concentrated by centrifugation. The final pellet was resuspended in 10 mM Hepes, 150 mM NaCl, pH 7.4 (hepes-sodium buffer), rapidly frozen in liquid nitrogen and stored at −80°C. For lipid composition analysis, samples were resuspended in methanol upon thawing and then assessed in a UPLC coupled to an orthogonal acceleration time-of-flight mass spectrometer with an electrospray ionization interface (LCT Premier; Waters) using the procedure previously described in [68]. Data were acquired using positive ionization mode over a mass range of m/z 50–1,500 in W-mode. A scan time of 0.15 s and interscan delay of 0.01 s were used at a nominal instrument resolution of 11,500 (FWHM). Leucine enkephalin was used as the lock spray calibrant.

### Primary Cell Cultures

Peripheral blood mononuclear cells (PBMCs) were obtained from HIV-1-seronegative donors and monocyte populations (>97% CD14^+^) were isolated with CD14^+^-positive selection magnetic beads (Miltenyi Biotec). DCs were obtained culturing these cells in the presence of 1,000 IU/ml of granulocyte-macrophage colony-stimulating factor (GM-CSF) and interleukin-4 (IL-4; R&D). mDCs were differentiated by culturing iDCs at day five for two more days in the presence of 100 ng/ml of lipopolysaccharide (LPS; Sigma). DCs were immunophenotyped at day 7 as previously described [15]. Adequate differentiation from monocytes to iDCs was based on the loss of CD14 and the acquisition of DC-SIGN, while DC maturation upregulated the expression of CD83, CD86, and HLA-DR. CD4^+^ T lymphocytes required for *trans*-infection experiments were isolated from PBMCs with CD4-negative selection magnetic beads (Miltenyi Biotec) and stimulated for 72 h in the presence of 10 IU/ml of IL-2 (Roche) and 3 µg/ml of phytohaemagglutinin (PHA; SigmaAldrich). Primary cells were maintained in RPMI with 10% fetal bovine serum (FBS), 100 IU/ml of penicillin and 100 µg/ml of streptomycin (all from Invitrogen).

### Cell Lines, Plasmids, and Viral Stocks

HEK-293T and TZM-bl (obtained through the US National Institutes of Health [NIH] AIDS Research and Reference Reagent Program, from JC Kappes, X Wu, and Tranzyme Inc.) were maintained in D-MEM (Invitrogen). CHO cell line was maintained in α-MEM. MT4 cell line was maintained in RPMI. All media contained 10% FBS, 100 IU/ml of penicillin, and 100 µg/ml of streptomycin. VLP_HIV-Gag-eGFP_ were obtained transfecting the molecular clone pGag-eGFP (obtained through the NIH AIDS Research and Reference Reagent Program, from MD Resh). VLP_MLV-Gag-YFP_ were obtained transfecting the molecular clone pGag-MLV wt [69]. HEK-293T cells were transfected with calcium phosphate (CalPhos, Clontech) in T75 flasks using 30 µg of plasmid DNA. CHO cells were electroporated (0.24 Kv and 950 µF) using 7×10^6^ cells and 40 µg of plasmid DNA. Supernatants containing VLPs were filtered (Millex HV, 0.45 µm; Millipore) and frozen at −80°C until use. For studies with concentrated VLPs, medium was harvested, cleared by filtration, and particles were concentrated by ultracentrifugation (28,000 rpm 2 h at 4°C in SW32 rotor) through 20% (w/w) sucrose. The final pellet was resuspended in hepes-sodium buffer, rapidly frozen in liquid nitrogen, and stored at −80°C. The p24^Gag^ content of the MT4-derived viral stock and VLP_HIV-Gag-eGFP_was determined by an ELISA (Perkin-Elmer) and by a quantitative Western blot. Detection was carried out with a LiCoR Odyssey system employing our own Rabbit anti-capsid polyclonal antibody and a purified Gag protein (kindly provided by J Mak) as a standard. To produce HIV_NL4-3_ in PBMCs, cells were stimulated with 3 µg/ml PHA and 10 IU/ml of IL-2 for 72 h prior to infection with HIV_NL4-3_. To generate HIV_NL4-3_ in CD4^+^ T cells, whole blood from three HIV-1–seronegative donors were CD8^+^ T cell depleted using Rossettesep anti-CD8^+^ cocktail (Stem cell). Enriched CD4^+^ T cells were pooled and stimulated under three different conditions: low-dose PHA (0.5 µg/ml), high-dose PHA (5 µg/ml), or plate-bound anti-CD3 monoclonal antibody OKT3 (e-Bioscience) [70]. After 72 h, cells were mixed together, infected with HIV_NL4-3_ and resuspended to a final concentration of 10^6^ cells/ml in RPM1 with 10% FBS supplemented with 100 IU/ml of IL-2. To produce glycosphingolipid deficient HIV_NL4-3_, enriched CD4^+^ T cells were kept in the presence or absence of 500 µM of NB-DNJ (Calbiochem) and 10 IU/ml of IL-2 for 6 d before stimulation with 3 µg/ml of PHA and subsequent infection with HIV_NL4-3_. Virus growth was monitored by p24^Gag^ ELISA (Perkin Elmer). Supernatants were harvested when the concentration of p24^Gag^ was at least 10^2^ ng/ml, filtered and stored at −80°C until use. Titers of all viruses were determined using the TZM-bl indicator cell line as described elsewhere [71].

### Production of Liposomes

LUVs were prepared following the extrusion method described in [72]. Lipids were purchased from Avanti Polar Lipids and gangliosides were obtained from Santa Cruz Biotechnology. Ganglioside source was bovine brain with the exception of GM4, which was from human brain. The LUV_HIV-tRed_ lipid composition was: POPC 25 mol%: 1,2-dipalmitoyl-sn-glycero-3-phosphocholine (DPPC) 16 mol%: brain sphingomyelin (SM) 14 mol%: cholesterol (Chol) 45 mol%, and when Cer, PS, or gangliosides were present (4 mol%) the SM amount was reduced to 10 mol%. The LUV_POPC-tRed_ lipid composition was 96 mol% POPC containing or not 4 mol% of Cer, GM3, GM2, or GM1a. All the LUVs contained 2 mol% of 1,2-dihexadecanoyl-sn-glycero-3-phosphoethanolamine (DHPE)-tRed (Molecular Probes). Lipids were mixed in chloroform∶methanol (2∶1) and dried under nitrogen. Traces of organic solvent were removed by vacuum pumping for 1–2 h. Subsequently, the dried lipid film was dispersed in hepes-sodium buffer and subjected to ten freeze-thaw cycles prior to extruding ten times through two stacked polycarbonate membranes with a 100-nm pore size (Nucleopore, Inc.) using the Thermo-barrel extruder (Lipex extruder, Northern Lipids, Inc.). In order to perform mDC pulse with equal concentrations of LUV displaying similar fluorescence intensities, tRed containing LUVs concentration was quantified following the phosphate determination method of [73] and the fluorescence emission spectra was recorded setting the excitation at 580 nm in a SLM Aminco series 2 spectrofluorimeter (Spectronic Instruments).

### Liposome and VLP Capture Assays

All capture experiments were performed pulsing mDCs in parallel at a constant rate of 100 µM of distinct LUV_tRed_ formulations and 75 ng of VLP_HIV-Gag-eGFP_ Gag quantified by western blot (2,500 pg of VLP_HIV-Gag-eGFP_ p24^Gag^ estimated by ELISA) per 2×10^5^ cells for 4 h at 37°C. After extensive washing, positive DCs were acquired by FACS with a FACSCalibur (BD) using CellQuest software to analyze collected data. Forward-angle and side-scatter light gating were used to exclude dead cells and debris from all the analysis.

Competition experiments were done incubating 2×10^5^ mDCs with 75 ng of VLP_HIV-Gag-eGFP_ Gag at a final concentration of 1×10^6^ cells/ml for 4 h at 37°C in the presence of decreasing amounts of GM2-containing LUV_HIV-tRed_ or 100 µM of Cer- and PS-containing LUV_HIV-tRed_. Alternatively, cells were incubated with 75 ng of VLP_HIV-Gag-eGFP_ Gag and 100 µM of LUV_HIV-tRed_ including or not GM1a, GD1b, GT1b, GQ1b, Cer, and PS. Cells were then analyzed by FACS as previously described.

### Confocal Microscopy Analysis

Co-localization experiments were done pulsing mDCs sequentially with LUV_HIV-tRed_ and VLP_HIV-Gag-eGFP_ for 3 h as described in the capture assays section. Capture patterns were analyzed similarly, incubating cells with distinct LUV_HIV-tRed_ and VLP_HIV-Gag-eGFP_ separately. After extensive washing, cells were fixed and cytospun into glass slides, mounted in DAPI-containing fluorescent media and sealed with nail polish to analyze them in a spinning disk confocal microscope. Z-sections were acquired at 0.1-µm steps using a 60× Nikon objective. Spinning disk confocal microscopy was performed on a Nikon TI Eclipse inverted optical microscope equipped with an Ultraview spinning disk setup (PerkinElmer) fitted with a two CCD camera (Hamamatsu). The co-localization signals in percentages of the compartment area and the thresholded Manders and Pearson coefficients were calculated for each image using Volocity 5.1 software (Improvision). To obtain 3-D reconstructions, confocal Z stacks were processed with Volocity 5.1 software, employing the isosurface module for the nucleus and the maximum fluorescent intensity projection for LUVs and VLPs.

### Neuraminidase Treatment of VLPs and LUVs

A total of 2×10^5^ DCs were pulsed for 2 h at 37°C with 25 µM of GM3-containing LUV_HIV-tRed_ and 75 ng of sucrose-pelleted VLP_HIV-Gag-eGFP_ Gag treated or not during 12 h at 37°C with 100 or 50 mU of neuraminidase from *Clostridium perfringens* Factor X (Sigma Aldrich). The 12-h incubation was done in a glass-coated plate (LabHut) in hepes-sodium buffer, and the reaction was stopped adding RPMI media containing FBS. Cells were washed and assessed by FACS to obtain the percentage of tRed- and eGFP-positive cells.

### Lactose and GM3 Polar Head Group Treatment of mDCs

mDCs were preincubated with or without 5 or 10 mM of lactose (Sigma-Aldrich) and soluble GM3 carbohydrate head group (Carbosynth) for 30 min at RT. Cells were then pulsed with 50 µM of GM3-containing LUV_HIV-tRed_ and 75 ng of sucrose-pelleted VLP_HIV-Gag-eGFP_ Gag for 2 h at 37°C, at a final concentration of 5 or 10 mM for the compounds tested. Cells were analyzed by FACS as described previously. For experiments with HIV_NL4-3_ generated in primary CD4^+^ T cells, mDCs were equally pre-incubated with lactose and the GM3 head group.

### Minimize Energy Structures of Gangliosides

Minimal energy structures in vacuum were computed using Chem3D Ultra software employing the MM2-force field and the steepest-descent algorithm. Minimum root mean square gradient was set to 0.1; minimum and maximum move to 0.00001 and 1.0, respectively.

### Dendritic Cell capture and *Trans*-infection of HIV_NL4-3_

mDCs and iDCs (5×10^5^ cells) were exposed to 30 ng p24^Gag^ of HIV_NL4-3_ obtained from stimulated PBMCs for 4 h at 37°C. Cells were washed thoroughly to remove unbound particles, lysed, and assayed for cell-associated p24^Gag^ content by an ELISA. mDCs (2.5×10^5^ cells) were exposed to 50 ng p24^Gag^ of HIV_NL4-3_ obtained from CD4^+^ T cells treated or not with NB-DNJ, incubated and assayed as previously described to detect the cell-associated p24^Gag^. Alternatively, mDCs (2.5×10^5^ cells) pre-incubated or not with GM3 or lactose as previously indicated were exposed to 90 ng p24^Gag^ of HIV_NL4-3_ obtained from CD4^+^ T cells and assayed equally. For *trans*-infection assays, mDCs were pulsed equally but with a constant multiplicity of infection (MOI) of 0.1, extensively washed and co-cultured in quadruplicate with the TZM-bl reporter cell line at a ratio of 10^4^∶10^4^ cells in the presence of 0.5 µM of saquinavir to assay luciferase activity 48 h later (BrightGLo luciferase system; Promega) in a Fluoroskan Ascent FL luminometer. Background values consisting of non-HIV-1 pulsed co-cultures were subtracted for each sample (mean background of 8.668 RLUs×100). *Trans*-infection to primary cells was performed similarly, co-culturing pulsed mDCs with activated primary CD4^+^ T cells for 48 h on a 96-well U-bottom plate without removal of unbound viral particles. Co-cultures were performed in the presence or in the absence of 0.5 µM of saquinavir. Infection of activated primary CD4^+^ T cells was detected with FACS, measuring the intracellular p24^Gag^ content within the CD2-positive CD11c negative population of CD4^+^ T cells employing the monoclonal antibodies p24^Gag^-FITC (KC57-FITC, clone FH190-1-1, Beckman Coulter), CD2-PerCP Cy5.5 (clone RPA-2.10, BD Pharmingen), and CD11c-APC Cy7 (clone Bu15, BioLegend). Co-cultures containing non-pulsed cells were used as a background control for p24^Gag^ labeling and used to set up the marker at 0.5%. To detect the possible cell free virus infection of activated CD4^+^ T cells, an equal MOI was added directly to control wells lacking mDCs.

### Statistical Analysis

Statistics were performed using GraphPad Prism v.5 software.

## Supporting Information

Figure S1 **Ganglioside structures.** 2-D model of asialo-, monosialo-, disialo-, trisialo-, and tetrasialo- gangliosides used in this study.
(PDF)

Figure S2 **Comparative fluorescence of tRed-containing LUVs.** Maximum emission fluorescence at 608 nm of LUV_HIV-tRed_ or LUV_POPC-tRed_ containing the molecules indicated in the graphs. (A) Comparison of LUV_HIV-tRed_ used in Figures 1 and 2; (B) comparison of LUV_POPC-tRed_ used in Figure 3; (C) comparison of LUV_HIV-tRed_ used in Figure 4; and (D) comparison of LUV_HIV-tRed_ used in Figure 5. Data show mean and SEM from independent measurements from at least two distinct LUV preparations.
(PDF)

Figure S3 **FACS capture-profiles of distinct LUVs in mDCs.** Histograms showing a representative capture-profile from Figure 1B, obtained pulsing mDCs derived from the same donor with 100 µM of distinct fluorescent LUV_HIV-tRed_ containing or not Cer, GM3, GM2, or GM1a for 4 h at 37°C.
(PDF)

Figure S4 **Polarized accumulation of ganglioside-containing LUVs and VLPs in mDCs.** Confocal microscopy analysis of mDCs previously pulsed with 100 µM of GM1a, GM2, and GM3 containing LUV_HIV-tRed_ and then exposed to 75 ng of VLP_HIV-Gag-eGFP_ Gag as in Figure 2B. 3-D reconstructions of the *x*-*y* sections collected throughout the whole mDC *z* volume every 0.1 µm. Isosurface representation of DAPI stained nucleus is shown, computing the maximum intensity fluorescence within a 3-D volumetric *x*-*y*-*z* data field, where VLP_HIV-Gag-eGFP_ and ganglioside-containing LUV_HIV-tRed_ polarized towards the same area of mDCs.
(PDF)

Figure S5 **Binding of distinct LUVs to mDCs.** (A) Comparative mDC binding of LUV_HIV-tRed_ containing GM3, Cer, or PS treated or not with *C. perfringens* neuraminidase for 12 h prior to addition to cells. A total of 2×10^5^ DCs were pulsed for 20 min at 37°C with 100 µM of LUV, washed with PBS, and assessed by FACS to obtain the percentage of tRed-positive cells. Data show mean values and SEM of cells from three donors. mDCs bound significantly higher amounts of untreated GM3 containing LUV_HIV-tRed_ than neuraminidase treated liposomes (*p* = 0.024, paired *t* test). (B) Binding pattern analysis of mDCs pulsed with LUV_HIV-tRed_ containing GM3, Cer, or PS treated or not with *C. perfringens* neuraminidase for 12 h prior addition to cells. Cells were incubated for 20 min at 37°C (top images) or 2 h at 16°C (bottom images) with100 µM of LUV, washed with PBS, and assessed by confocal microscopy. After 20 min at 37°C, liposomes remained randomly bound and no evident polarization or internalization was detected, as seen in mDCs incubated at 16°C to arrest endocytosis. Images show 3-D reconstruction of the *x*-*y* sections collected throughout the whole mDC *z* volume every 0.1 µm, computing the maximum intensity fluorescence of the liposome red signal and DAPI-stained nucleus.
(PDF)

Figure S6 **Minimal energy structures of the different gangliosides tested.** Blue shadow indicates the proposed sialyllactose viral attachment moiety recognized by mDCs. For comparative purposes, GM4 and Asialo GM1 lacking sialyllactose domains are also depicted.
(PDF)

Video S1 **3-D reconstruction of an mDC pulsed with GM3 containing LUV_HIVtRed_ and then exposed to VLP_HIV-Gag-eGFP_.** Confocal microscopy analysis of an mDC pulsed with GM3 containing LUV_HIVtRed_ and then exposed to VLP_HIV-Gag-eGFP_ as in Figure 2b. The video shows a 3-D reconstruction of the *x*-*y* sections collected throughout the whole mDC *z* volume every 0.1 µm. Isosurface representation of DAPI stained nucleus is depicted, computing the maximum intensity fluorescence of the sac-like compartment surface within a 3-D volumetric *x*-*y*-*z* data field, where VLP_HIV-Gag-eGFP_ and GM3-containing LUV_HIV-tRed_ are accumulated within the same compartment.
(MOV)

## Abbreviations

Cer: ceramide
DC: dendritic cell
FACS: fluorescence activated cell sorting
FBS: fetal bovine serum
eGFP: enhanced GFP, green fluorescent protein
iDC: immature dendritic cell
IL: interleukin
LUV: large unilamellar vesicle
mDC: mature dendritic cell
MOI: multiplicity of infection
PBMC: peripheral blood mononuclear cell
PHA: phytohaemagglutinin
POPC: 1-palmitoyl-2-oleoyl-sn-glycero-3-phosphocholine
PS: phosphatidylserine
SEM: standard error of the mean
tRed: Texas Red
VLP: virus-like particle
