# Supplementary figures and images for: Sialyllactose in Viral Membrane Gangliosides Is a Novel Molecular Recognition Pattern for Mature Dendritic Cell Capture of HIV-1
Source: PLoS Biol. 2012 Apr 24;10(4):e1001315. doi: 10.1371/journal.pbio.1001315 (PMC3335875; doi:10.1371/journal.pbio.1001315)

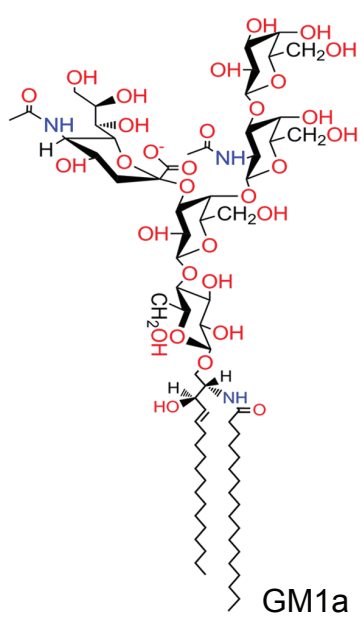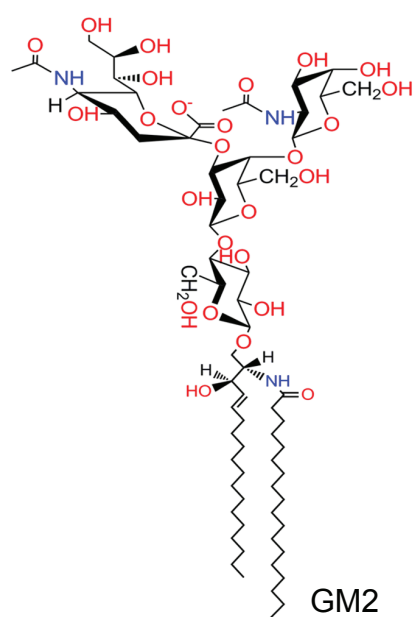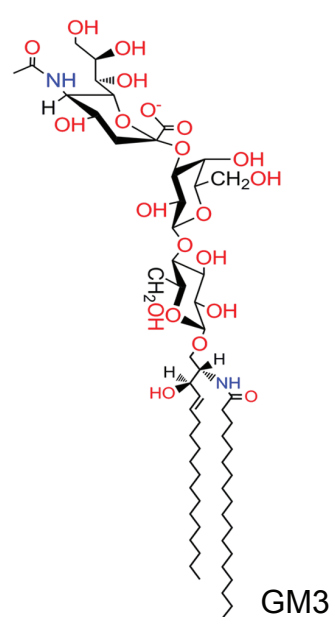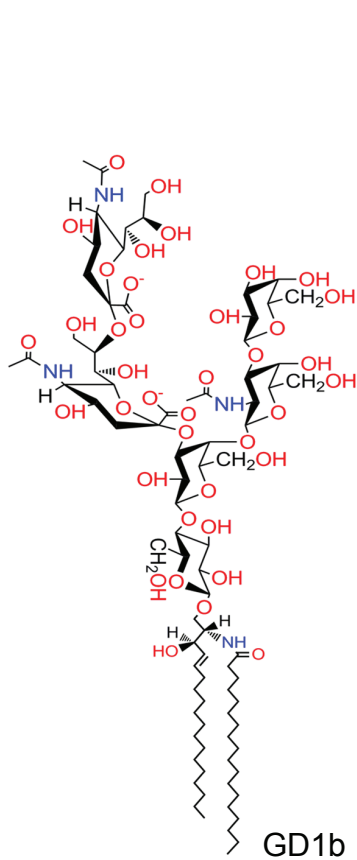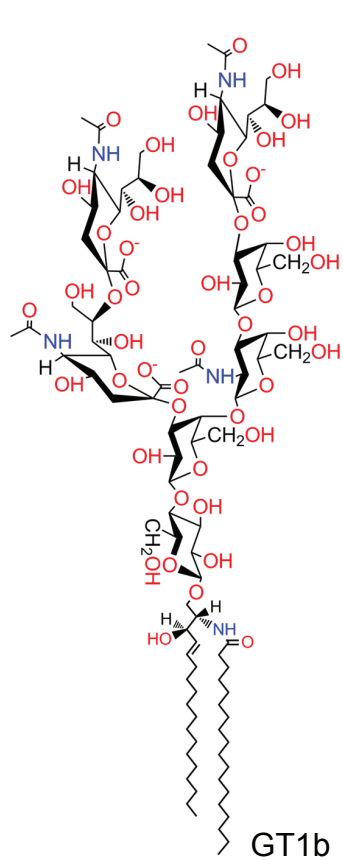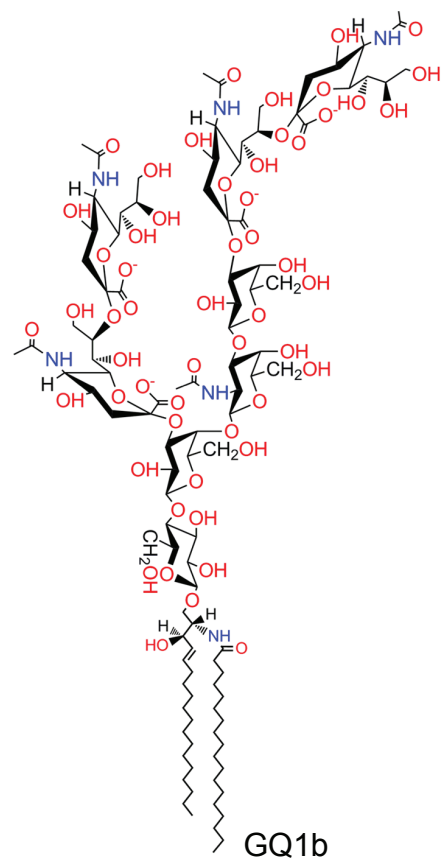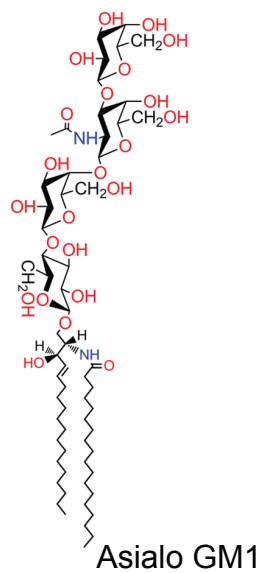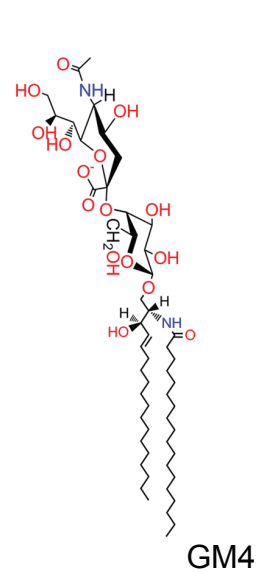

Supplement: Figure S1 — Ganglioside structures. 2-D model of asialo-, monosialo-, disialo-, trisialo-, and tetrasialo- gangliosides used in this study. (PDF) [file pbio.1001315.s001.pdf]

A

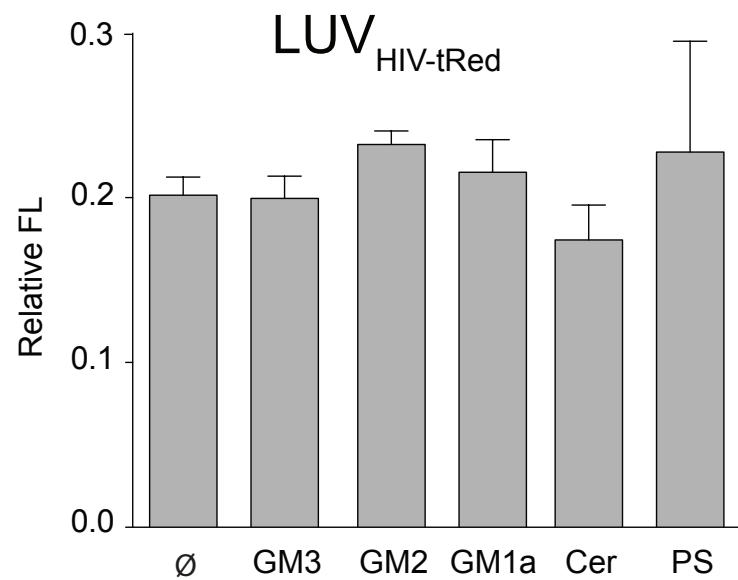

B

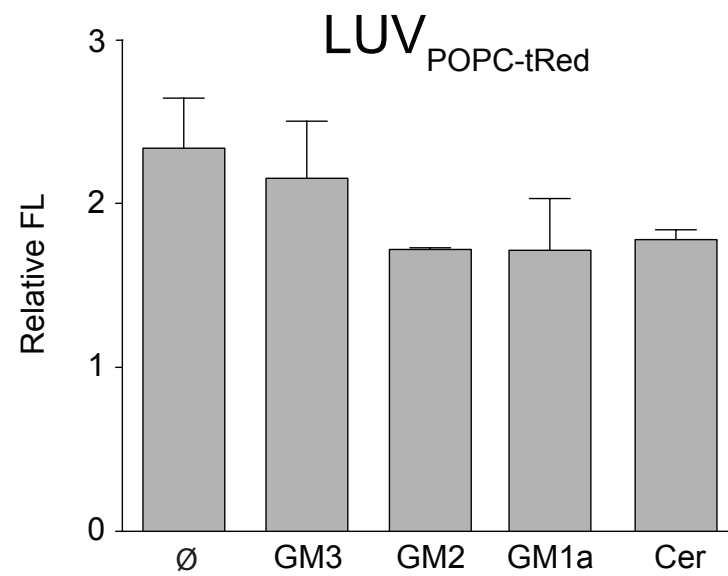

C

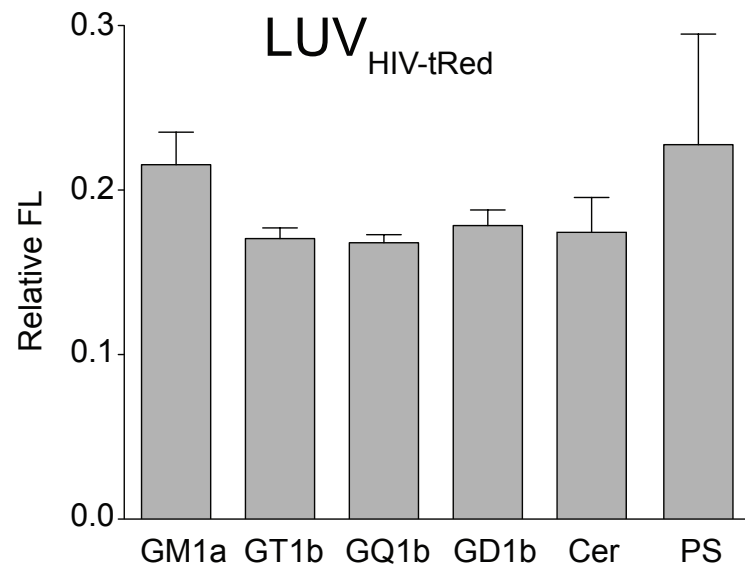

D

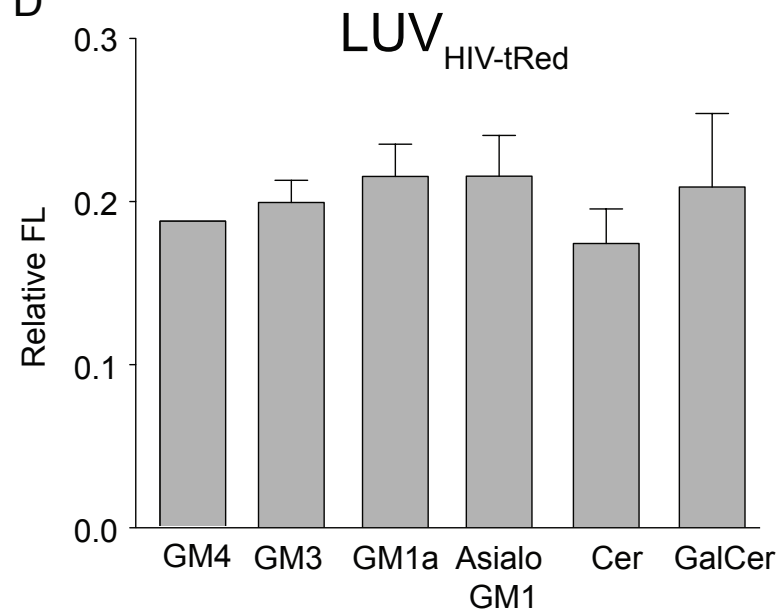

Supplement: Figure S2 — Comparative fluorescence of tRed-containing LUVs. Maximum emission fluorescence at 608 nm of LUVHIV-tRed or LUVPOPC-tRed containing the molecules indicated in the graphs. (A) Comparison of LUVHIV-tRed used in Figures 1 and 2; (B) comparison of LUVPOPC-tRed used in Figure 3; (C) comparison of LUVHIV-tRed used in Figure 4; and (D) comparison of LUVHIV-tRed used in Figure 5. Data show mean and SEM from independent measurements from at least two distinct LUV preparations. (PDF) [file pbio.1001315.s002.pdf]

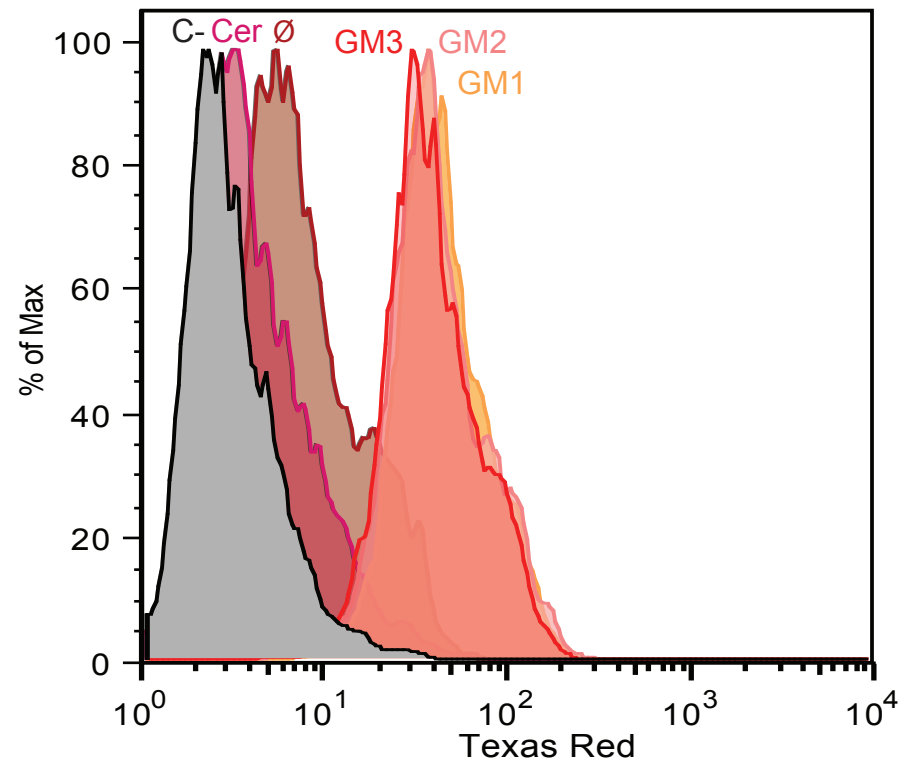

Supplement: Figure S3 — FACS capture-profiles of distinct LUVs in mDCs. Histograms showing a representative capture-profile from Figure 1B, obtained pulsing mDCs derived from the same donor with 100 µM of distinct fluorescent LUVHIV-tRed containing or not Cer, GM3, GM2, or GM1a for 4 h at 37°C. (PDF) [file pbio.1001315.s003.pdf]

LUV<sub>HIV-tRed</sub>+GM3

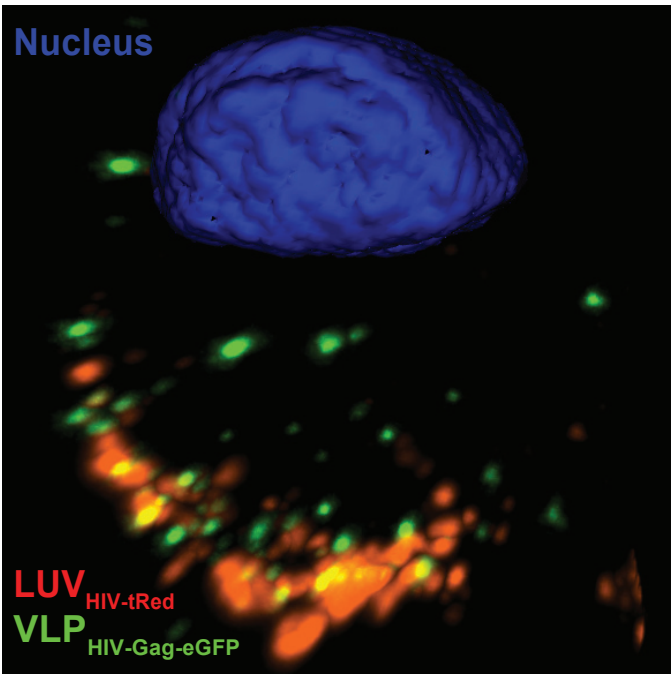

LUV<sub>HIV-tRed</sub>+GM2

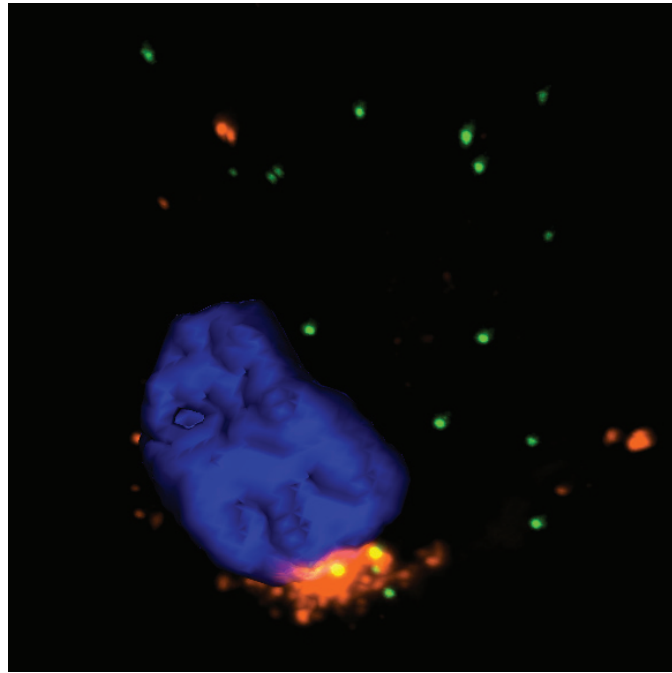

LUV<sub>HIV-tRed</sub>+GM1a

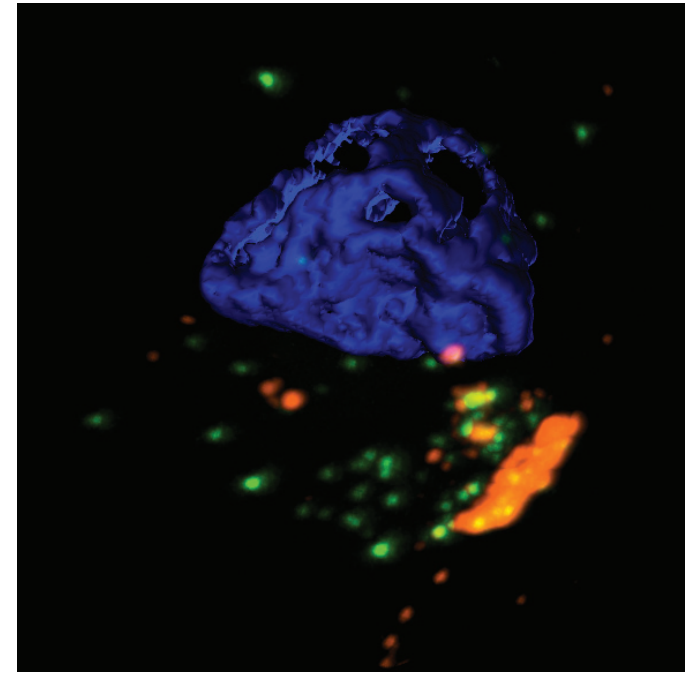

Supplement: Figure S4 — Polarized accumulation of ganglioside-containing LUVs and VLPs in mDCs. Confocal microscopy analysis of mDCs previously pulsed with 100 µM of GM1a, GM2, and GM3 containing LUVHIV-tRed and then exposed to 75 ng of VLPHIV-Gag-eGFP Gag as in Figure 2B. 3-D reconstructions of the x-y sections collected throughout the whole mDC z volume every 0.1 µm. Isosurface representation of DAPI stained nucleus is shown, computing the maximum intensity fluorescence within a 3-D volumetric x-y-z data field, where VLPHIV-Gag-eGFP and ganglioside-containing LUVHIV-tRed polarized towards the same area of mDCs. (PDF) [file pbio.1001315.s004.pdf]

A

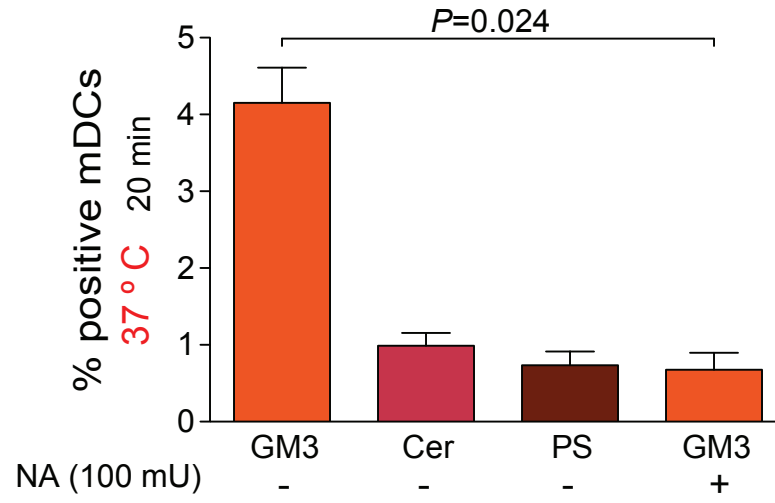

B

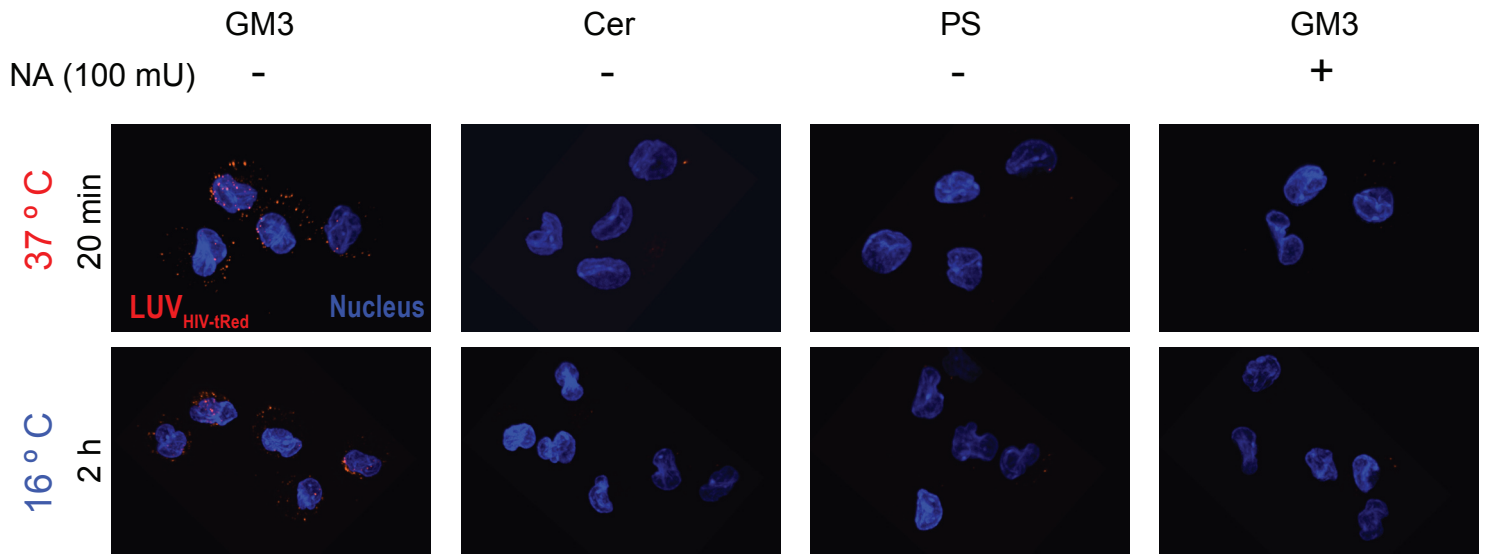

Supplement: Figure S5 — Binding of distinct LUVs to mDCs. (A) Comparative mDC binding of LUVHIV-tRed containing GM3, Cer, or PS treated or not with C. perfringens neuraminidase for 12 h prior to addition to cells. A total of 2×10^5^ DCs were pulsed for 20 min at 37°C with 100 µM of LUV, washed with PBS, and assessed by FACS to obtain the percentage of tRed-positive cells. Data show mean values and SEM of cells from three donors. mDCs bound significantly higher amounts of untreated GM3 containing LUVHIV-tRed than neuraminidase treated liposomes (p = 0.024, paired t test). (B) Binding pattern analysis of mDCs pulsed with LUVHIV-tRed containing GM3, Cer, or PS treated or not with C. perfringens neuraminidase for 12 h prior addition to cells. Cells were incubated for 20 min at 37°C (top images) or 2 h at 16°C (bottom images) with100 µM of LUV, washed with PBS, and assessed by confocal microscopy. After 20 min at 37°C, liposomes remained randomly bound and no evident polarization or internalization was detected, as seen in mDCs incubated at 16°C to arrest endocytosis. Images show 3-D reconstruction of the x-y sections collected throughout the whole mDC z volume every 0.1 µm, computing the maximum intensity fluorescence of the liposome red signal and DAPI-stained nucleus. (PDF) [file pbio.1001315.s005.pdf]

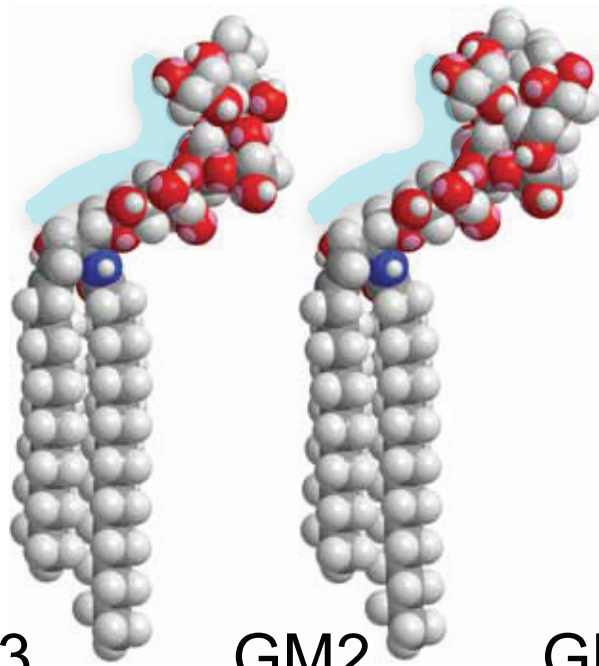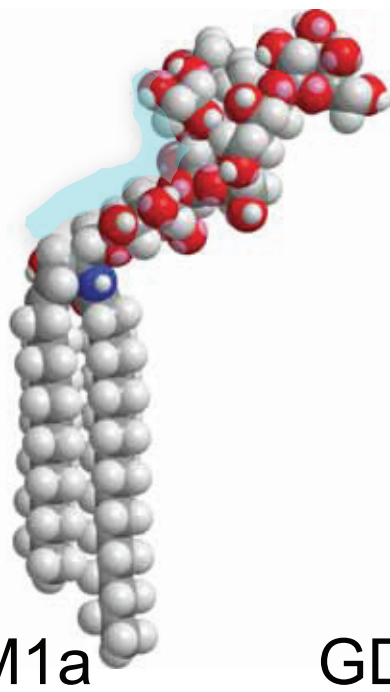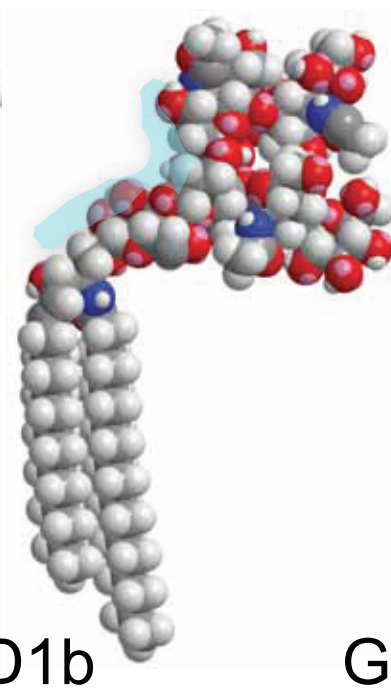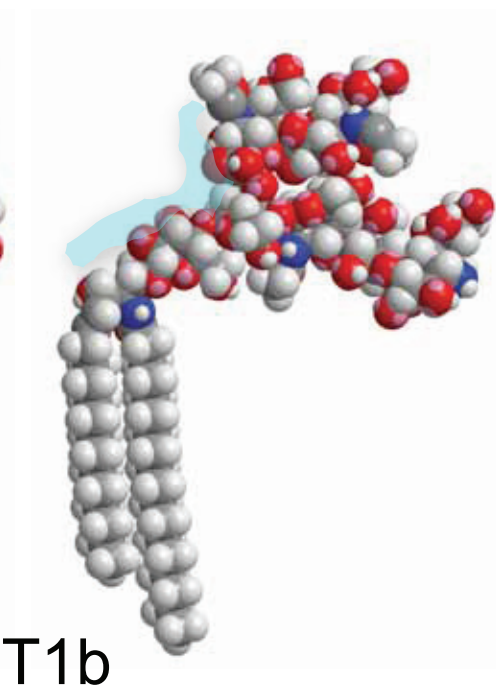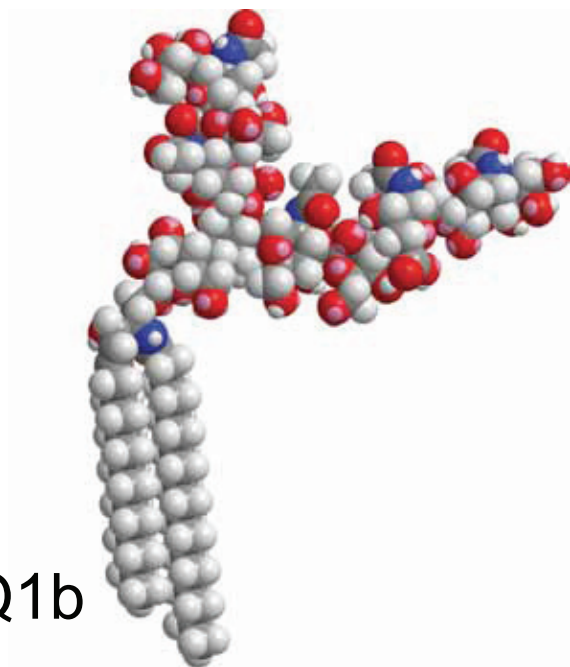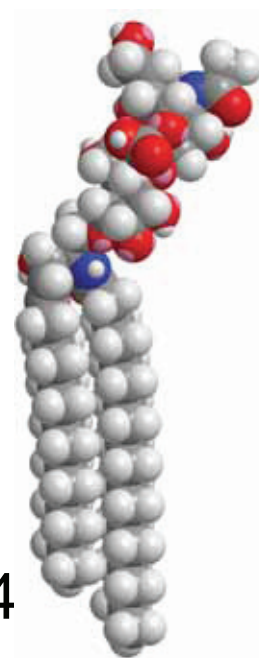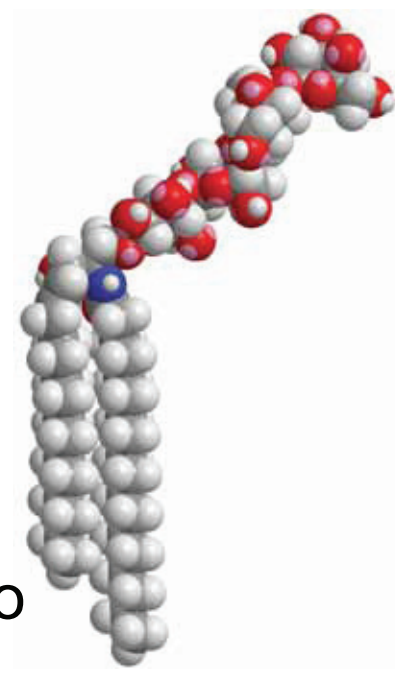

Supplement: Figure S6 — Minimal energy structures of the different gangliosides tested. Blue shadow indicates the proposed sialyllactose viral attachment moiety recognized by mDCs. For comparative purposes, GM4 and Asialo GM1 lacking sialyllactose domains are also depicted. (PDF) [file pbio.1001315.s006.pdf]
